# Neural Network Based Contact Force Control Algorithm for Walking Robots

**DOI:** 10.3390/s21010287

**Published:** 2021-01-04

**Authors:** Byeongjin Kim, Soohyun Kim

**Affiliations:** Department of Mechanical Engineering, KAIST, Daejeon 34141, Korea; geniuskpj@kaist.ac.kr

**Keywords:** neural network, push-off, walking, force control, contact force, ground reaction force

## Abstract

Walking algorithms using push-off improve moving efficiency and disturbance rejection performance. However, the algorithm based on classical contact force control requires an exact model or a Force/Torque sensor. This paper proposes a novel contact force control algorithm based on neural networks. The proposed model is adapted to a linear quadratic regulator for position control and balance. The results demonstrate that this neural network-based model can accurately generate force and effectively reduce errors without requiring a sensor. The effectiveness of the algorithm is assessed with the realistic test model. Compared to the Jacobian-based calculation, our algorithm significantly improves the accuracy of the force control. One step simulation was used to analyze the robustness of the algorithm. In summary, this walking control algorithm generates a push-off force with precision and enables it to reject disturbance rapidly.

## 1. Introduction

**Figure 1:**
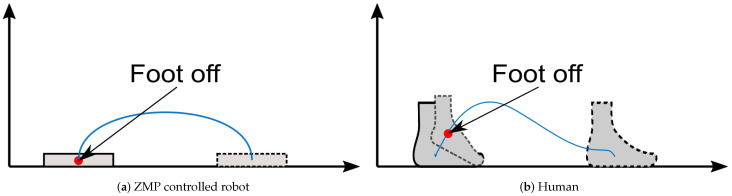
Swing leg’s foot trajectory of (**a**) robot and (**b**) human during gait cycle. Robot’s trajectory is from pre-developed robot [25]. Human gait is running at 3.56 m/s [26,27].

Zero Moment Point (ZMP) control is widely accepted as a basic approach for walking robots [1,2,3,4,5,6,7,8,9,10]. Kajita [11] suggested a preview control system based on the Cart–Table model to generate biped walking patterns by ZMP. The preview control model requires the Center of Mass (CoM)’s height and weight to predict a stable CoM trajectory with the desired ZMP. However, the preview controller is not the most ideal for feedback because ZMP contains acceleration terms and also real-time feedback control in the influence of external disturbances is challenging. As described in Figure 1a, a ZMP-controlled robot swings its leg in order to take off and touches the leg down softly to the pre-defined footstep placement. When accelerating, a ZMP-controlled robot only uses its stance leg to accelerate, whereas humans utilize the propulsive push-off power from the swinging leg to accelerate [12,13] as shown in Figure 1b. The reason why the push-off mechanism is not used for ZMP-controlled robots is because push-off makes ZMP perturbation. Ground Reaction Forces (GRFs) from push-off are considered a disturbance, which is one of potential reasons for the efficiency gap between humans and robots [14,15,16,17]. Therefore, here we propose a new control method by push-off that does not cause ZMP perturbation [18].

Atrias [19,20] is a bipedal walking robot co-developed by Carnegie Mellon University and Oregon State University. The developers of Atrias have shown that Series Elastic Actuator (SEA) and Spring Loaded Inverted Pendulum (SLIP) model could improve the efficiency of walking. Atrias achieved a 9 km/h speed and robust walking on an uneven terrain with human like GRF. However, because GRF is controlled directly by the SLIP model, Atrias cannot stand still without making constant motion. Therefore, this solution is not applicable for robots that need to operate at a standstill. The researchers of Atrias calculated a required joint torque using the Jacobian method without using mass terms of foot.

A more complicated model does not guarantee a better performance because the uncertainties are coming from the model. Lee [21] used a null space method to regulate the GRF and kept the robot’s balance. The null space method could control effectively without a Force/Torque (F/T) sensor. Moreover, the performance is modulated by inertia information. Park [22] proposed a hybrid approach for contact force control combining the null space method and observer. The approach removes the effect of disturbance or error with the F/T sensor [23] by applying a filter or observer to reduce noise from sensed signal. Furthermore, the observer can estimate the force for sensorless control [24]. However, these approaches have delays that significantly reduce the performance and stability of the robots. the F/T sensor for bipedal robots is also high cost and weighs over 500 g.

Neural network (NN) learning is a useful method for unknown complicated models [28,29]. NN-based hybrid position/force control was proposed by Passold [30] and Kumar [31]. Moreover, observer based approaches have been reported in [32,33,34]. However, these researches also used a force sensor in their model. Unlike sensor based control, Xu [35] suggested an impedance control based on an observer without force sensor. Adaption algorithm is applied to estimate stiffness and position of environment. Yet, this model has a significant delay for early contact.

In this paper, we propose a NN model to control ground reaction force without F/T sensor. This approach reduces modeling error and effort for finding model parameters. The contributions of our work are shown as follows:

- We propose a model based on neural network (NN) estimation on push-off force (GRF) control. It effectively decreases errors created by the robot’s mass and gravity.
- The neural network model is applied with Linear Quadratic Regulator (LQR), which is adopted for balancing and generating desired force for push-off.
- We introduce the simulation to prove that our approach is validated in dynamic situations like walking.

The rest of the paper is organized as follows. Section 2 introduces the NN model and procedure of the simulation. Section 3 shows The results obtained from our model and simulation. The analysis about the result is summarized in Section 4. Section 5 concludes the paper.

## 2. Method

### 2.1. Neural Network Model

**Figure 2:**
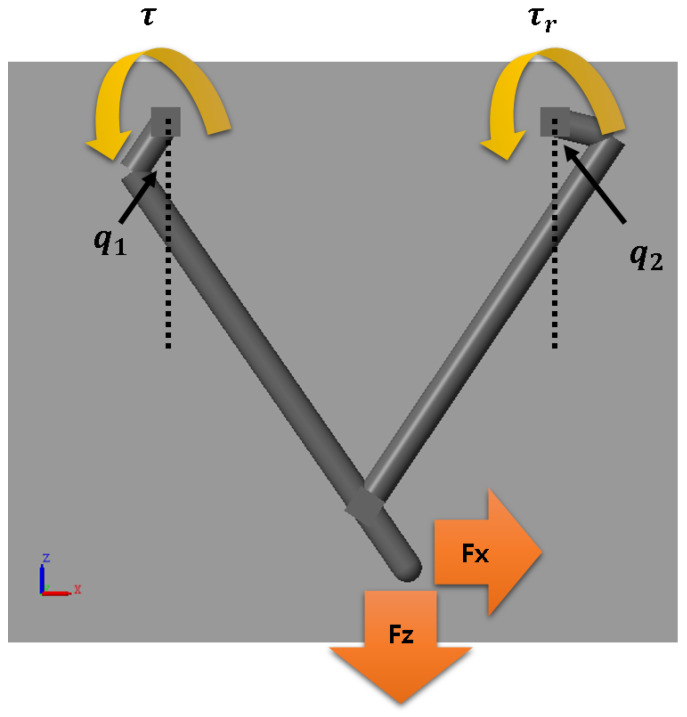
Leg model for neural network regression.

The parallel type leg in this paper is shown in Figure 2. It is developed for reducing inertia and fast walking. Physical modeling and simulation are executed on the MATLAB Simscape. There are two motors on the hip to control the position (x,z) of the foot. The hip is connected to the body with a roll motor and is fixed during the NN training.

Dynamic equation for leg can be formulated as:

(1) $$I\ddot{\theta }+C\left(\theta ,\dot{\theta }\right)+K\theta +G\left(\theta \right)+J^{T}F=\tau$$

where *I* is the inertia matrix, *C* represents the coriolis and centrifugal term, *K* is the stiffness matrix and G(θ) is the gravity function. The matrix *J* is the contact Jacobian for the contact position and *F* is the contact force matrix. θ is the joint angle matrix and τ is the joint torque matrix. If we the know exact model, the reference torque for the desired force is obtained by:

(2) $$\tau_{reference}=I{\ddot{\theta }}_{m}+C\left(\theta_{m},{\dot{\theta }}_{m}\right)+K\theta_{m}+G\left(\theta_{m}\right)+J^{T}F_{desired}$$

where $\theta_{m}$ represents the measured joint angle. Because the model information from CAD is not perfect, we need to conduct experiments for calculating the exact parameter [18]. Besides, links must be decomposed, and the test takes much time and effort. Furthermore, if we use a force sensor or observer to compensate the modeling error, the delay is unavoidable.

Thus, we propose a neural network model for removing model error without force sensor. Because the inertia of the leg is negligible against the body in this model, *I*, *C* could be omitted. Then, the reference torque is simplified by:

(3) $$\tau_{reference}=K\theta_{m}+G\left(\theta_{m}\right)+J^{T}F_{desired}=h\left(q_{1},q_{2},F_{dx},F_{dz}\right)$$

where $q_{1},q_{2}$ are the joint angle from the left and right motors, $F_{dx},F_{dz}$ represents the x-axis and z-axis of the desired contact force. We assume the robot’s foot is fixed on a vertically moving stage with a force sensor. When the torque $\left(\tau ,\tau_{r}\right)$ is applied on the foot position by the joint angle $\left(q_{1},q_{2}\right)$, we could measure the generated force $F_{x},F_{z}$.

Our purpose is to acquire an approximation function of *h* by using these data. The neural network is efficient for curve fitting and works well in nonlinear regression. To avoid overfitting, the Bayesian regularization algorithm is adopted.

**Figure 3:**
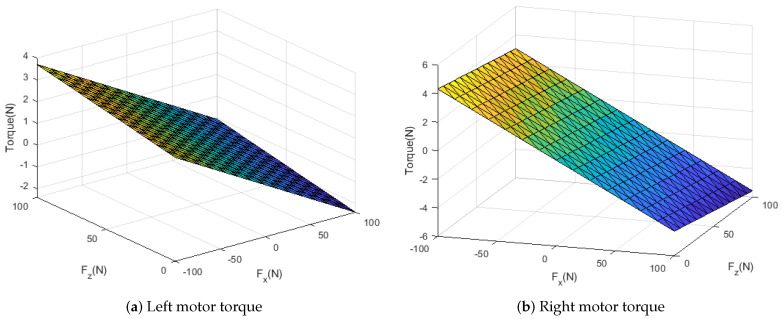
Measured torque is plotted for each force, $q_{1}=-\frac{\pi }{14},q_{2}=\frac{\pi }{14}$.

**Figure 4:**
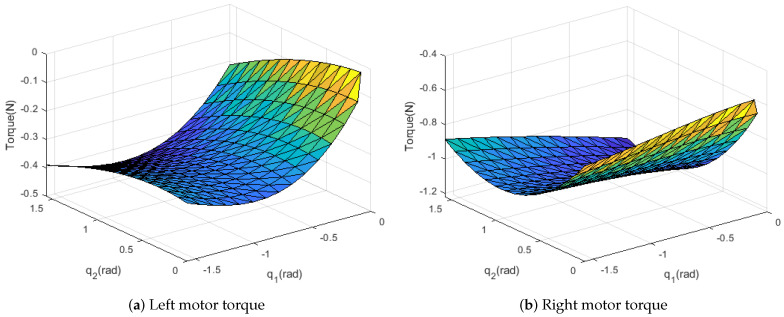
Measured torque is plotted for each joint angle, $F_{x}=\frac{100}{7}N,F_{z}=\frac{100}{7}N$.

In Figure 3 and Figure 4, the torque surface is plotted to determine the neural network parameter. The figures show *h* is not a highly nonlinear function. Therefore, we used one hidden layer. When the data are divided into 10 values in the range, sufficient performance is achieved. 10,000 datasets are collected under each $10q_{1}\times 10q_{2}\times 10F_{x}\times 10F_{z}$. Performance by random datasets is not significantly different. Ranges of input data are determined as follows:

$$F\in \left[-100,100\right]N,q_{1}\in \left[-\frac{\pi }{2},0\right]rad,q_{2}\in \left[0,\frac{\pi }{2}\right]rad$$

**Figure 5:**
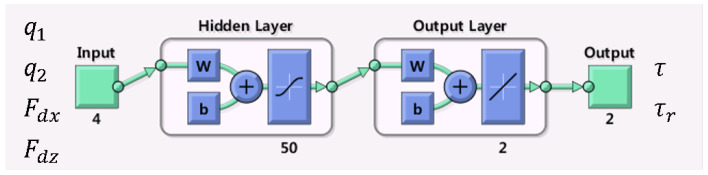
Neural network model for regression.

The structure of the NN model is described in Figure 5 and in Equation (4).

(4) $$y=b_{2}+w_{L}*tansig\left(b_{1}+w_{I}x\right)$$

- *y* = output vector = ${\left[\begin{array}{cc} \tau & \tau_{r} \end{array}\right]}^{T}$.
- $b_{1},b_{2}$ = bias vector.
- $w_{L}$ = Layer weight matrix, $w_{I}$ = Input weight matrix.
- $tansig\left(n\right)=\frac{2}{1+e^{-2n}}-1$.
- *x* = input vector = ${\left[\begin{array}{cccc} q_{1} & q_{2} & F_{dx} & F_{dz} \end{array}\right]}^{T}$.

We used 70% of the data for training, 5% for validation, and 25% for the test. The parameters for NN learning are shown in Table 1. Training stops when the maximum number of epochs is reached, μ exceeds $\mu_{max}$ or the gradient falls below min_grad.

#### Cad Model

The same approach is tested on a realistic CAD model. It contains a motor, gearbox and it is fully designed for manufacturing. We assume this leg has the same workspace as a pre-developed robot [25]. The leg length is twice as long. The motor is selected for an increased length and weight. One motor position is changed because the motor is too big to place side by side. The range of $q_{1}$ is not symmetric because the length of the left and right link are not the same.

$$F_{x}\in \left[-200,200\right]N,F_{z}\in \left[-100,1000\right]N,q_{1}\in \left[0.82q_{2}-0.6067,0.82q_{2}+0.52\right]rad,q_{2}\in \left[0,1.8\right]rad$$

### 2.2. Lqr Design

**Figure 6:**
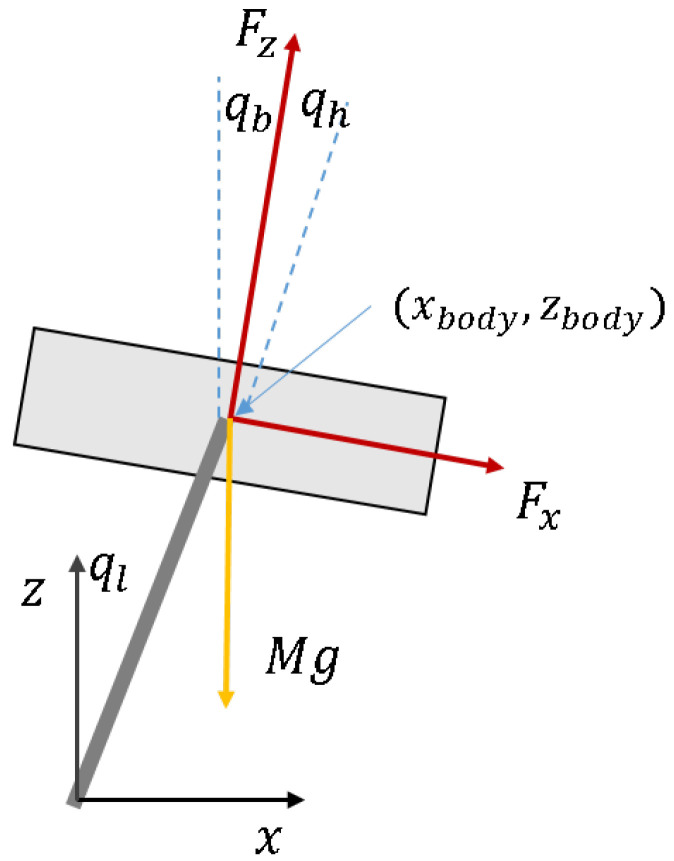
Free body diagram for body.

Figure 6 shows the 2D robot model and the parallel leg is simplified into a single effective link. LQR is an optimal feedback controller for a linear plant, and is easily tuned for multiple objectives by state (Q) and input (R) weight matrix.

The equations obtained from the model are shown below:

(5) $$\begin{array}{c} \left\{\begin{array}{cc} M\ddot{x} & =F_{x}cos\left(q_{b}\right)\\ M\ddot{z} & =F_{z}cos\left(q_{b}\right)-Mg\;\mathrm{for}\;\mathrm{body} \end{array}\right.\\ \left\{\begin{array}{cc} R_{x} & =-F_{x}cos\left(q_{b}\right)\\ R_{z} & =Mg-F_{z}cos\left(q_{b}\right)\;\mathrm{for}\;\mathrm{leg}\\ q_{b} & =q_{l}-q_{h}\\ I\ddot{q_{l}} & =R_{x}lcos\left(q_{l}\right)-R_{z}lsin\left(q_{l}\right) \end{array}\right. \end{array}$$

- *M* = mass of robot, *g* = gravity acceleration.
- *F* = force from leg to body.
- *R* = force from body to leg.
- $q_{b}$ = body angle, $q_{h}$ = hip angle, $q_{l}$ = leg angle.
- *I* = inertia of robot, *l* = length from body to foot.

Besides high gain being applied in the support phase, we assume *l* is constant. The mass of the leg is $\frac{1}{20}$ scale of robot mass, thus it is ignored. At a linearized point, $q_{b}^{🟉}=q_{l}^{🟉}\approx 0$. The state-space equation for LQR can be written as:

(6) $$\begin{array}{cc} H\dot{X} & =AX+BU\\ \dot{X} & =H^{-1}\left(AX+BU\right)=A^{\prime }X+B^{\prime }U\\ X & ={\left[\begin{array}{cccccc} q_{b} & \dot{q_{b}} & x & \dot{x} & z & \dot{z} \end{array}\right]}^{T}\\ H & =diag(\left[\begin{array}{cccccc} 1 & I & 1 & M & 1 & M \end{array}\right])\\ A & =\left[\begin{array}{cccccc} 0 & 1 & 0 & 0 & 0 & 0\\ 0 & 0 & 0 & 0 & 0 & 0\\ 0 & 0 & 0 & 1 & 0 & 0\\ 0 & 0 & 0 & 0 & 0 & 0\\ 0 & 0 & 0 & 0 & 0 & 1\\ 0 & 0 & 0 & 0 & 0 & 0 \end{array}\right]\\ B & =\left[\begin{array}{cc} 0 & 0\\ -l & 0\\ 0 & 0\\ 1 & 0\\ 0 & 0\\ 0 & 1 \end{array}\right]\\ U & ={\left[\begin{array}{cc} F_{x} & F_{z} \end{array}\right]}^{T} \end{array}$$

We use simple PD control on hip roll motors.

(7) $$u_{roll}=K_{P}\left(q_{ref}-q_{roll}\right)-K_{D}{\dot{q}}_{roll}$$

$q_{roll}$ = angle of roll motor, ${\dot{q}}_{roll}$ = angular velocity of roll motor, $q_{ref}$ = reference angle

$q_{ref}$ is a constant that keeps initial hip roll angle.

(8) $$\begin{array}{cc} K_{lqr} & =\left[\begin{array}{cccccc} 100 & 10 & 50,000 & 5000 & 0 & 0\\ 0 & 0 & 0 & 0 & 50,000 & 5000 \end{array}\right]\\ U & =-K_{lqr}X \end{array}$$

$K_{lqr}$ is calculated from (A,B,Q,R) by the algebric Riccati equation. The controllability matrix is not full rank but is stabilizable. It means that the robot could fall from some initial states. Controlling *x* and $q_{b}$ are trade-offs because two states are coupled. If we give too much weight to *x*, $q_{b}$ is not controlled properly. After some simulation, we could find appropriate ratio for them.

The entire control flow is described as:

1. LQR calculates desired contact force ($F_{x},F_{z}$) from error.
2. Desired Joint torque ($\tau ,\tau_{r}$) is obtained from a neural network regression model.
3. Apply the joint torque to the robot plant and measure the states.

**Figure 7:**
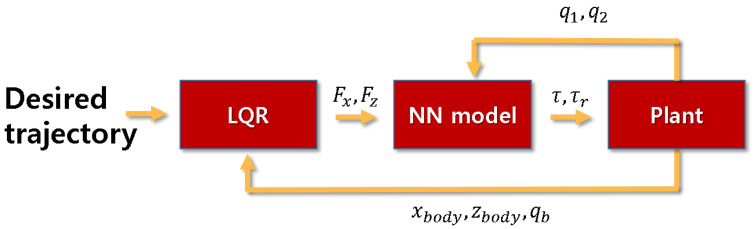
Entire control flow, NN means neural network model.

Workflow is also described in Figure 7.

### 2.3. One Step Simulation

**Figure 8:**
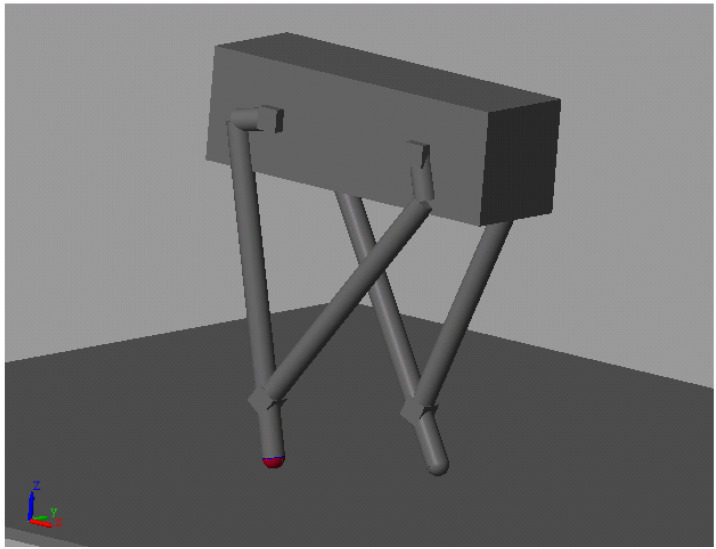
Robot model for simscape.

**Figure 9:**
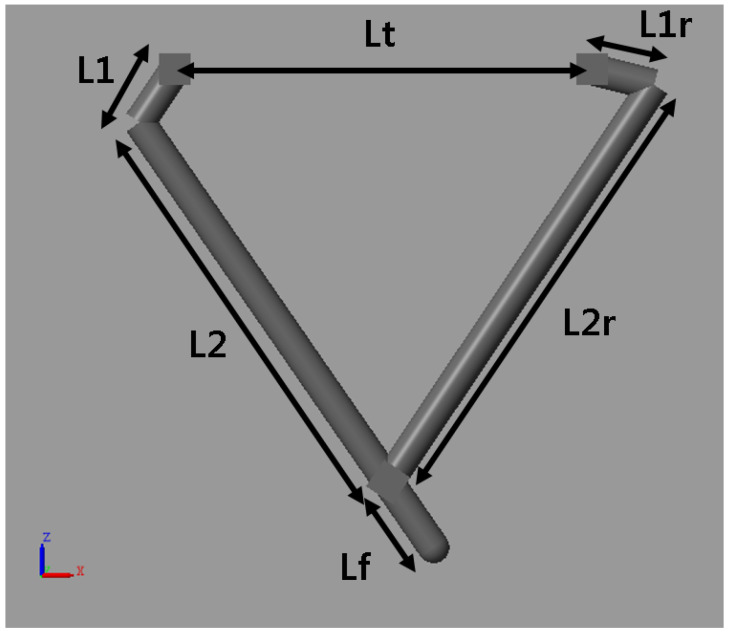
Leg model parameter.

We establish a simulation model to verify that the proposed approach works well while the robot body is moving. The robot model is demonstrated in Figure 8. There are two hip roll motors on the invisible frame. The control objective for the hip roll motor is to sustain the angle. There is no external constraint on the model, but only spatial contacts between the spherical foot and ground. The static friction coefficient is 1 and the dynamic is 0.8. This is the friction coefficient of rubber on dry concrete. The hip width is 0.1m and the other parameters are written in Figure 9 and Table 2.

Leg length parameters are optimized for minimizing the required torque, and the step width is 0.1 m. The push-off force is generated by trajectory based on the trigonometric function ($p_{desired}=\frac{A}{2}\left(1-cos\left(\frac{2\pi t}{T}\right)\right)$). The maximum force for the z-axis is 170% of the mass and force for the x-axis is 40%. The step time and amplitude of the push-off is selected by tuning.

Simulation starts from the double support phase. Right leg push-off ground and mode is changed into a single support phase. Body angle and effective leg length *l* are regulated in a single support phase. After heel-strike, the robot is balanced.

## 3. Results

### 3.1. Neural Network Model

We did the test to prove robustness:

1. Pick random desired force.
2. Calculate the joint torque by using a neural network model.
3. Compare the real torque for the desired force with a calculated one (Test 1).
4. Apply the joint torque and compare the measured force with the desired (Test 2).

**Figure 10:**
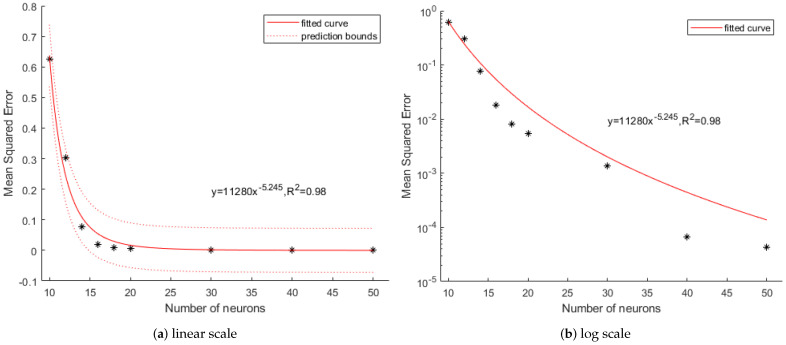
Mean square error of torque by the number of neurons.

Figure 10 shows the logarithmic MSE decrease as the number of neurons increases. However, neurons more than 50 show negligible performance improvement in force generation (Test 2). Thus, we decide the number of neurons is 50 and it needs about 27 ms to calculate torque.

**Figure 11:**
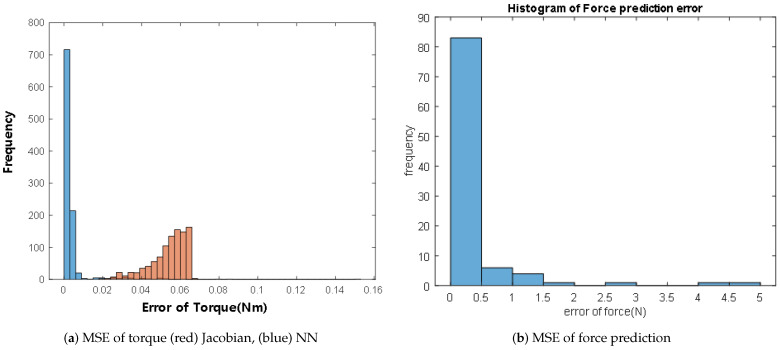
Histogram of mean square error.

Test 1 executed 1000 times and Test 2 executed 100 times. When we use the Jacobian method, torque is calculated by $\tau_{reference}=J^{T}F_{desired}$. The gravitational term is not applied because we assume the mass model is not accurate. In the error histogram (Figure 11a), the torque error from the NN model is much lower than the error from the Jacobian method. Table 3 shows the predicted torque error by dismissing *I*, *C*. In Test 2, the mean error from the Jacobian method is 4.58 N and the mean error from NN is 0.37 N. The mean error is reduced by 92% when a neural network model is applied.

#### CAD Model

**Figure 12:**
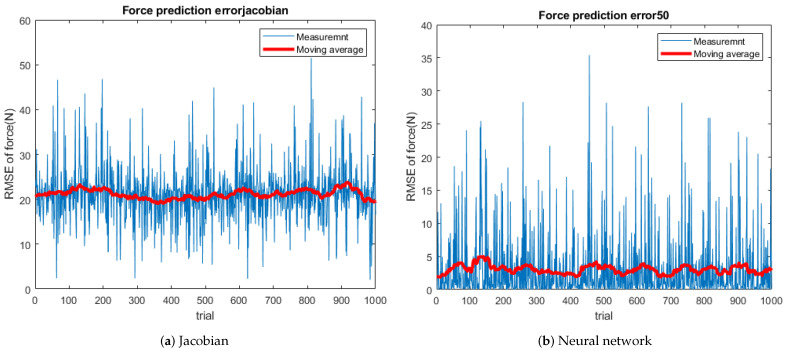
Force prediction error.

To prove the robustness of the NN model, Test 2 is applied to the CAD model. The root mean squared error (RMSE) from the NN model is 73.7% lower than the Jacobian method. Because the CAD model is heavier (4.5 kg) than simplified model (300 g), Figure 12 shows increased force error. The moving average line indicates the error from NN is significantly smaller than the Jacobian’s.

**Figure 13:**
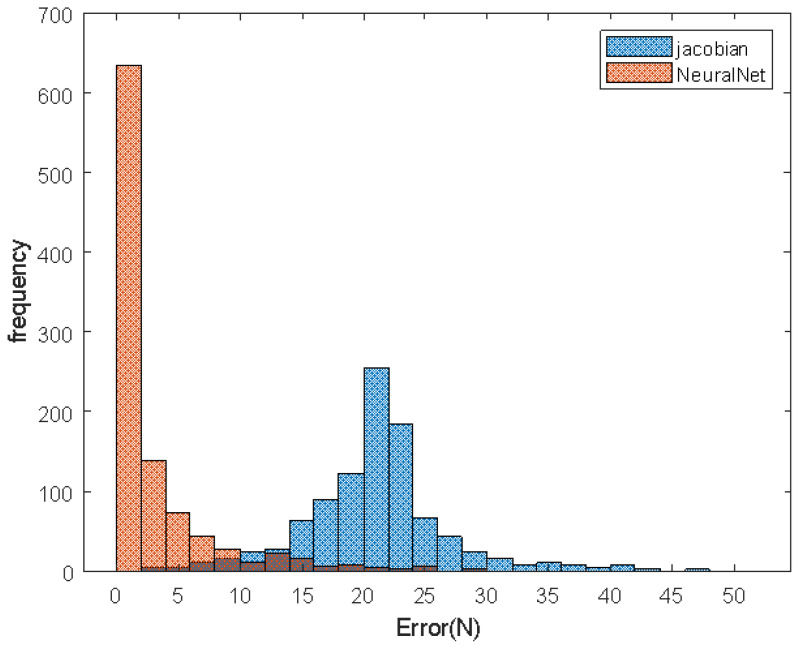
Histogram of force prediction error.

According to Figure 13 and Table 4, the average error is reduced by 85.6%. There are some spikes over 10 N in the NN model. When the leg is almost fully stretched, the spikes appear by a singularity. Simscape solver’s linearization and approximation also make an error.

### 3.2. One Step Simulation

**Figure 14:**
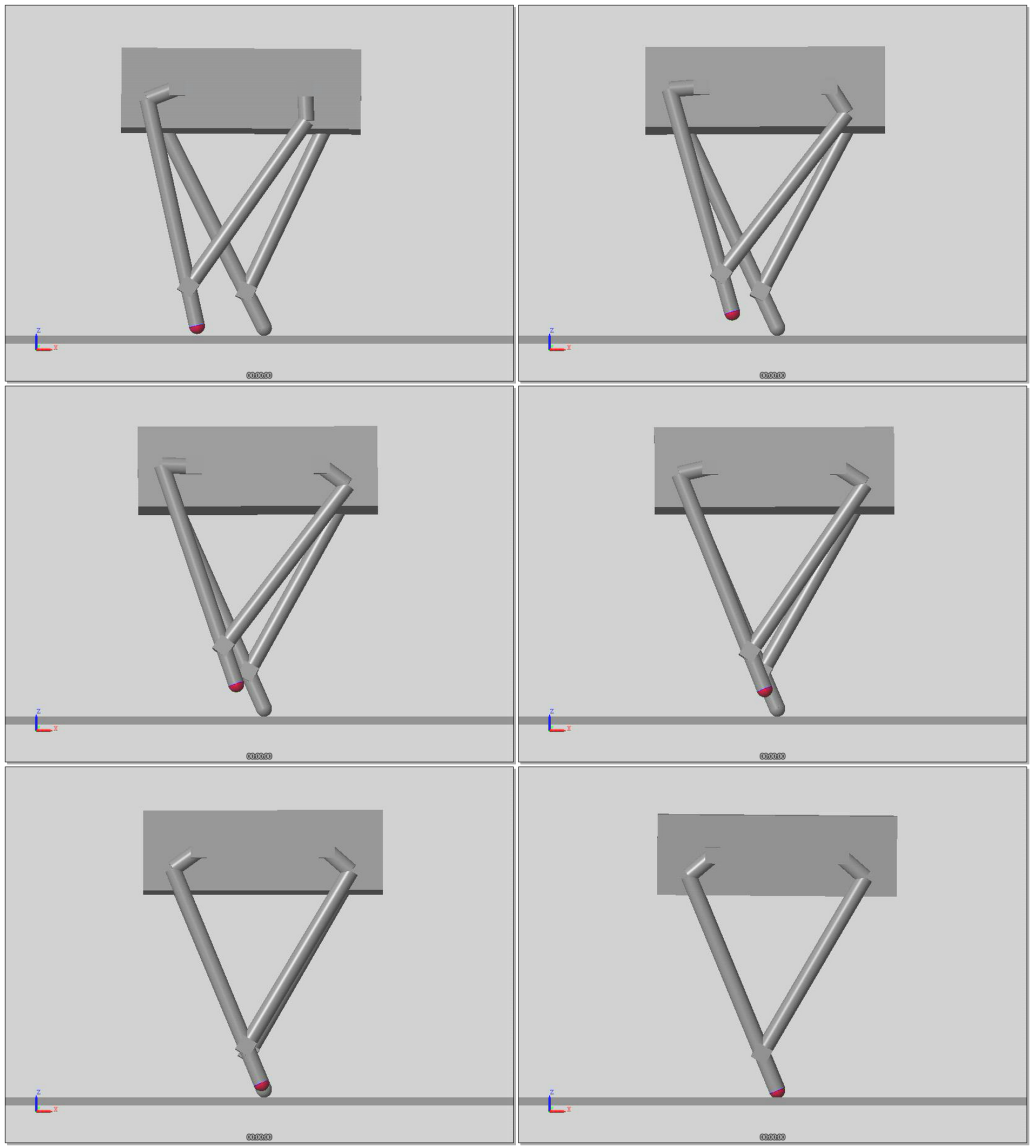
Still shot of one step simulation.

**Figure 15:**
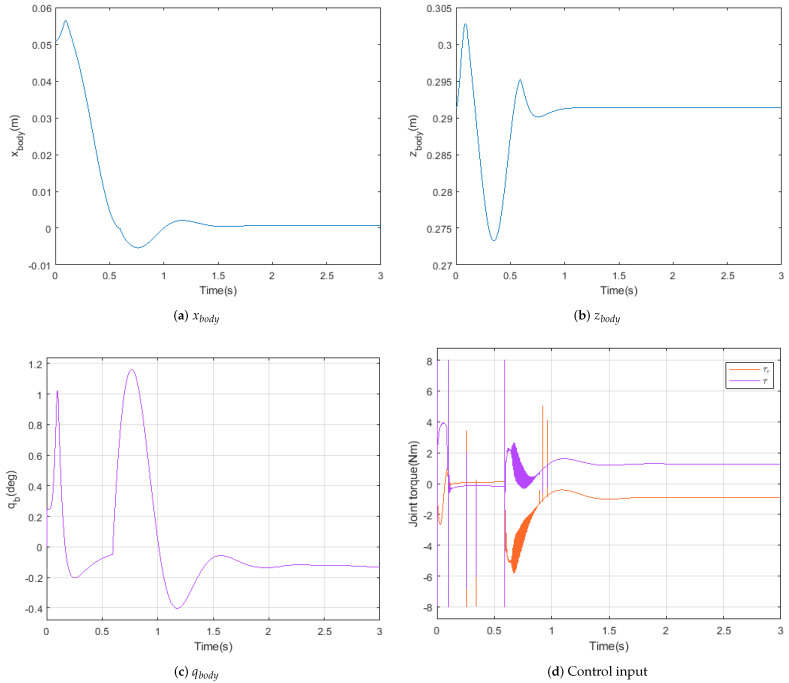
States and input of swing foot.

Figure 14 demonstrates the procedure of one step simulation. Figure 15 indicates the states and input of the LQR controller. Heel strike occurs at 0.6 s and the robot keeps balance without additional stepping. On the other hand, Atrias has no ability to keep balance at standstill [20]. It is critical for robots with manipulator.

Figure 15a,b show the movement of the body, taking maximum overshoot values of 1 cm and 2 cm. Figure 15c shows the response of the body angle $q_{b}$, which takes values between −0.4 and 1.2 degrees. The joint torque of the swing foot, having maximum values of −8 and 8 Nm, are presented in Figure 15d. Peak values appeared by mode transition and numerical error of simulation.

**Figure 16:**
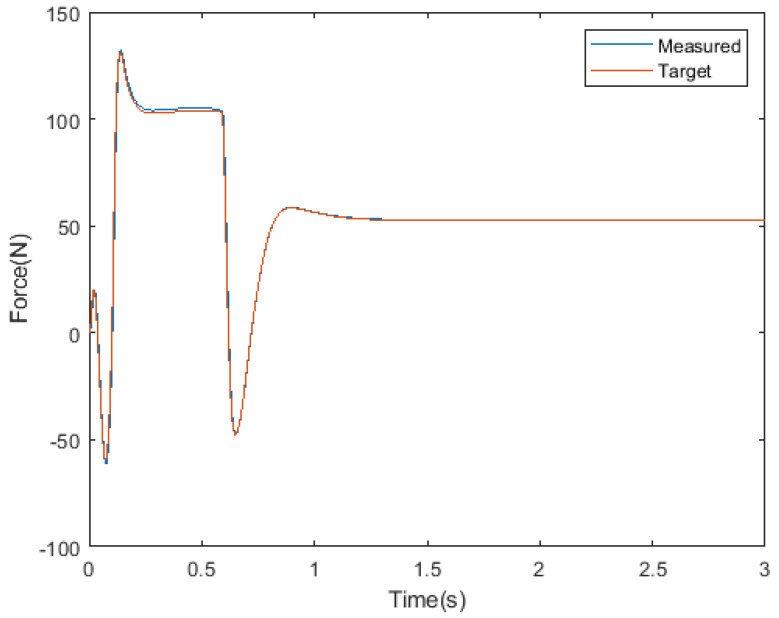
Measured and desired force.

Figure 16 shows the real and target normal force of the stance leg. The NN model operates normally under moving conditions. Average error is 0.64 N.

**Figure 17:**
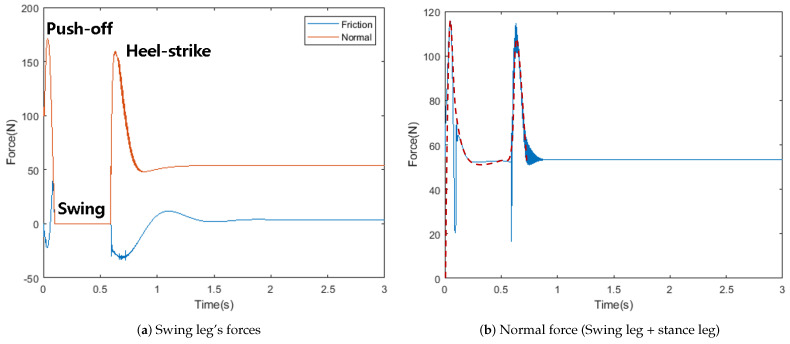
Normal and friction force of one step simulation. Red dashed line: approximation of sum.

In Figure 17a, GRF is not measured in the swing phase. Human’s GRF is drawn for a stance leg from heel strike to push-off [36]. It needs more steps to draw the grf of the stance leg. Hence, we draw similar results by adding the swing and stance leg’s normal force.

## 4. Discussion

The neural network model has a high accuracy in force control. The average and RMS error are smaller than 10 N. According to Table 4, the proposed model achieves better accuracy than the Jacobian method without including mass information. The NN model removes the time-consuming process of formulating equations and finding the parameters of each link. In biped robots, the error is produced by bolts, bearings, wire and electronic parts. Even though all terms are modeled, force control error always exists [21]. NN modeling is a systematic approach to modify models by experiments and learning. The main reason for performance improvement comes from dismissing gravitational terms in the Jacobian method. Since we designed the leg for faster movement, the leg mass under 5 Kg was used. There will be more improvement for heavier legs. In the manipulator’s case, a computed torque method or observer based approach is applied for better performance and removing the model error. Due to the noisy value from sensors, a filter or observer is used in those approaches. Stability and performance deteriorate because the delay is accompanied by filters. Unlike manipulators, walking robots could fall by a small delay. F/T sensors for an observer are also expensive and heavy.

Since Equation (3) has no terms about ground, the NN model is not affected by the condition and material of the ground. Therefore, the model is also working in a floating state. Figure 3 shows the model is linear about $F_{z}$. Thus, $F_{z}$ range for the dataset could be from 0 to positive value. If we get data only for pushing ground, it does not have any performance drop.

Based on the results in Section 3.2, our approach can be used for moving platforms. Figure 17b shows an approximated line that resembles a human’s GRF. There is a flat part between two peaks in the single support phase. Because the robot’s speed is 0.25 m/s which is much slower than a human’s (1–1.5 m/s), using push-off, the robot could achieve more speed for multiple step. Although push-off and heel strike are disturbances over 100 N, the proposed LQR controller rejects the disturbance with a small error. Robots could stand without any motion or additional stepping. ZMP-controlled robots usually need an F/T sensor or advanced control like a capture point to remove disturbance.

## 5. Conclusions

The purpose of this article is to provide a new contact force control algorithm. Unknown disturbance and modeling error will cause the failure of the walking robot. We propose a NN based force control method to solve these problems. We use NN regression to remove time-consuming steps like formulating equations and finding parameters for model. Secondly, we introduce the LQR walking controller with the NN model for balancing and push-off.

The performance of contact force generation was compared with the Jacobian method. The result shows that the neural network model significantly improves the performance of force control. The research pointed out that the proposed method is highly applicable for contact force control. Our approach has strong robustness on one step simulation which contains transient disturbance. The proposed algorithm satisfies requirements under simulation and the result shows the feasibility of applying our approach to walking control.

NN modeling can be easily implemented by other manipulators or robots. The advantage of these solutions can be exploited well on complicated multi-axis robots or parallel manipulators.

In our future work, we will add frictional or damping terms in the model. Friction modeling is a challenging problem to be added to force control. However, this is essential to improve performance in walking of real robots. The redundant degree of freedom will be considered later. Additional objects like torque minimization can be added to the model.

## Figures and Tables

**Table 1:** Paratmeters used for neural network training.

Parameters	Value	Description
epochs	1000	Maximum number of epochs
μ	0.005	Marquardt adjustment parameter
$\mu_{dec}$	0.1	Decrease factor for μ
$\mu_{inc}$	10	Increase factor for μ
$\mu_{max}$	$1\times 10^{10}$	Maximum value for μ
min_grad	$1\times 10^{-7}$	Minimum performance gradient

**Table 2:** Leg model parameter table.

Name	Value
L1 (m)	0.04
L1r (m)	0.04
L2 (m)	0.24
L2r (m)	0.255
Lf (m)	0.05
Lt (m)	0.08
*M* (kg)	10

**Table 3:** Statistical value of prove test.

	Test 1 (Nm)	Test 2 (N)
MSE	$9.45\times 10^{-5}$	0.67
Mean	0.0039	0.37
STD	0.0089	0.74

**Table 4:** Statistical value of prove test in CAD model.

Error	Max (N)	Avg (N)	Var (N)	RMSE (N)
Jacobian	51	21.2	36.2	22.0
Neural	36	3.1	24.2	5.8
Change (%)	−29.4	−85.6	−33.1	−73.7

## Data Availability

Data available on request due to restrictions by funding organization.

## Abbreviations

The following abbreviations are used in this manuscript:

NN	Neural Network
GRF	Ground Reaction Force
CoM	Center of Mass
LQR	Linear Quadratic Regulator
F/T	Force Torque
CAD	Computer Aided Design
ZMP	Zero Moment Point
